# Hypomineralized primary teeth and their association with Molar Incisor Hypomineralization: a cross-sectional study

**DOI:** 10.1590/1807-3107bor-2025.vol39.063

**Published:** 2025-06-02

**Authors:** Bruna Cordeiro AMARANTE, Leticia Yumi ARIMA, Giovanna Bueno MARINHO, Ana Carolina Cheron GENTILE, Edgard MICHEL-CROSATO, Marcelo BÖNECKER

**Affiliations:** (a) Universidade de São Paulo – USP, School of Dentistry, Department of Orthodontics and Pediatric Dentistry, São Paulo, SP, Brazil.; (b) Universidade de São Paulo – USP, School of Dentistry,Department of Public Health, São Paulo, SP, Brazil.

**Keywords:** Breast Feeding, Developmental Defects of Enamel, Molar Hypomineralization

## Abstract

Environmental and systemic factors play a role in the development of hypomineralization characterized by demarcated opacities. This study aimed to investigate the prevalence, distribution, severity, and etiologic factors associated with hypomineralization in all primary teeth, hypomineralized second primary molars (HSPM) and evaluate its association with Molar Incisor Hypomineralization (MIH). A sample of 2,102 male and female Brazilian children aged 3 to 10 years exhibiting good general health was examined by 30 calibrated dentists using WHO probes and clinical mirrors, using the index proposed by Ghanim et al. Data were collected through clinical examinations and a questionnaire answered by mothers, addressing sociodemographic and prenatal, perinatal, and postnatal factors. Prevalence was assessed by calculating frequency, whereas etiologic factors were evaluated using univariate and multivariate Poisson logistic regression with robust adjustment. In addition, multivariate analysis was conducted using nonparametric resampling with Jacknife adjustment. The overall prevalence of hypomineralization in primary teeth was 18.5%, with second molars affected in 17% and canines in 6.7% of the cases, while other teeth were affected at lower rates. Exclusive breastfeeding for six months showed a protective effect against hypomineralization (p = 0.40). Children with HSPM were five times more likely to develop MIH, regardless of sex (OR: 4.92). Furthermore, lower family income increased the likelihood of MIH. In conclusion, hypomineralization in primary teeth is prevalent, exclusive breastfeeding for six months is associated with a lower prevalence of enamel defects, and children with primary dentition defects are at greater risk for similar conditions in their permanent teeth.

## Introduction

Hypomineralization characterized by demarcated opacities has been increasingly diagnosed and studied.^1^ Hypomineralization are defects that affect the quality of enamel, characterized by demarcated opacities in shades of white, cream, yellow, and brown. It can affect both primary and permanent teeth.^2^ In primary dentition, second molars and canines are the most commonly affected teeth, a condition known as hypomineralized second primary molars (HSPM). In permanent dentition, first molars and incisors are the most affected teeth, and this condition is known as Molar Incisor Hypomineralization (MIH).^2^

The etiologic factors of hypomineralization, characterized by demarcated opacities in primary and permanent teeth, are still being investigated, and it is believed that a combination of environmental and systemic factors from the prenatal, perinatal, and postnatal periods and genetic factors may play a role.^3^ According to international studies, the prevalence of HSPM varies widely from 0% to 21.8%.^4,5^ The prevalence of HSPM in Brazil was reported in three studies: 20.1% in Minas Gerais,^6^ 6.5% in Brasília,^7^ and 14.9% in Piauí.^8^

According to the literature, the most frequently investigated factors for the prenatal period include maternal smoking,^9^ drug use,^10^ alcohol consumption,^9^ maternal diseases, and medication use during pregnancy.^11^ Associated factors for the perinatal period include prematurity,^8,12^ low birth weight, and complications during childbirth.^2^ Factors associated with the postnatal period are asthma,^8^ fever in the first year of life, respiratory tract infection, pneumonia,^12^ exclusive breastfeeding for less than 6 months,^2,11^ diarrhea, digestive disorders, kidney failure, ear infection, rubella, and chickenpox.^8-10,14^

An important aspect of the prevalence of HSPM is that its presence in young children’s oral cavity can be a predictor of MIH in the permanent dentition.^6,7^ This possibility arises from the fact that factors associated with the development of these enamel defects can occur during the first year of life, simultaneously affecting both dentitions, because mineralization of primary teeth finishes when mineralization of permanent teeth begins.^6,7^ Epidemiological studies have demonstrated that children with HSPM are six times more likely to develop MIH.^2,13-15^

Given the need for further research in this area, the present study aims to investigate the prevalence of hypomineralization, characterized by demarcated opacities in primary teeth, to assess the etiologic factors associated with it and evaluate the association between HSPM and MIH.

## Methods

The study was approved by the local research ethics committee (process no. 3.683.832) and conducted in accordance with the Declaration of Helsinki. The study followed the STROBE checklist for observational studies. The children’s parents or guardians signed an informed consent form, and literate children signed an assent form to take part in the study.

The study was conducted in Diadema, São Paulo, Brazil. Sample size was calculated considering the following data: size of the population of the municipality for the studied age group (23,000), sampling error of 2%, and 95% confidence interval, resulting in 2,097 participants. This study had a representative sample of 2,102 children in the specified age group, randomly selected and evenly distributed across local primary healthcare centers (PHCs), age groups, and sexes. Participants were included from October 2019 to March 2020. The study included male and female children aged 3 to 10 years exhibiting good general health who had been evaluated during oral health follow-up visits or had been screened at the PHC of Diadema. Children who did not cooperate during clinical examination or who wore fixed orthodontic appliances were excluded because of limited access to all tooth surfaces.

Hypomineralization in primary and permanent teeth was diagnosed using the criteria established by Ghanim et al.,^16^which assess eruption status, clinical features (absence or presence of the defect and its characteristics), and the extent of the enamel defect. Children were invited to attend one of the PHCs according to the follow-up oral health program for the collection of demographic, socioeconomic, and clinical data. The child was positioned in a dental chair for clinical examination. The examination was carried out under artificial lighting, using gauze to dry and clean the teeth, wooden spatulas, clinical mirrors and a WHO probe. The intraoral physical examination was performed systematically by quadrant and the clinical findings were entered by the dentist or dental assistant into a clinical form especially developed for this study.

Examinations were performed by 30 dentists who worked at the PHCs. All dentists were calibrated according to Amarante et al.^17^ Examiners’ kappa values were calculated and interpreted according to the values proposed by Landis and Koch.^28^ The average kappa value was 0.93 ± 0.07 for clinical criteria, 0.98 ± 0.08 for eruption criteria, and 0.75 ± 0.14 for extension criteria.

**Figure 1:**
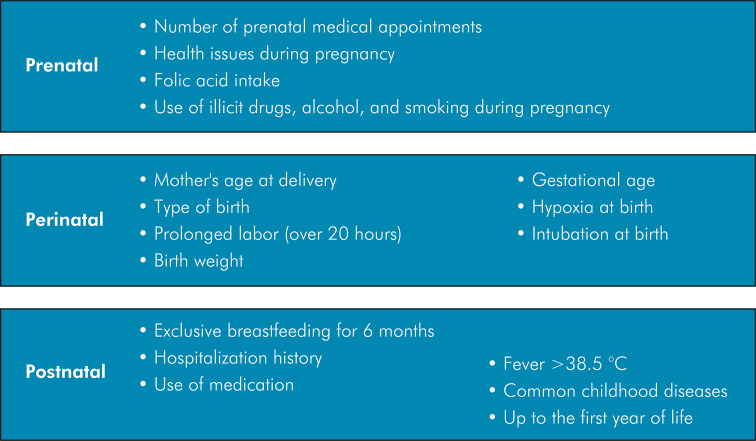
Etiologic factors associated with hypomineralization in primary teeth.

A questionnaire was designed to collect personal data from the children and their families and data on possible etiologic factors that could be associated with the presence of hypomineralization in primary teeth (Figure 1). Mothers were interviewed and answered the questionnaire. To reduce the risk of recall bias, only mothers were allowed to answer the questionnaire.

The variables included in the questionnaire were taken from the literature that indicated possible factors associated with the development of enamel defects. The studied variables related to the prenatal period were the number of prenatal medical appointments, health issues during pregnancy,^11^ folic acid intake,^11^ use of illicit drugs,^10^ and alcohol and smoking during pregnancy.^9^ The studied variables related to the perinatal period were gestational age at birth (in weeks),^8,12^ hypoxia at birth, intubation at birth, mother’s age at delivery, type of birth,^2^, prolonged labor (over 20 hours),^2^ and birth weight.^2^ The studied variables related to the postnatal period, up to one year of age were exclusive breastfeeding for 6 months,^2,12^ hospitalization history, use of medication,^8-10^ fever higher than 38.5°C,^12^ and common childhood diseases.^8-10,14^

**Figure 2:**
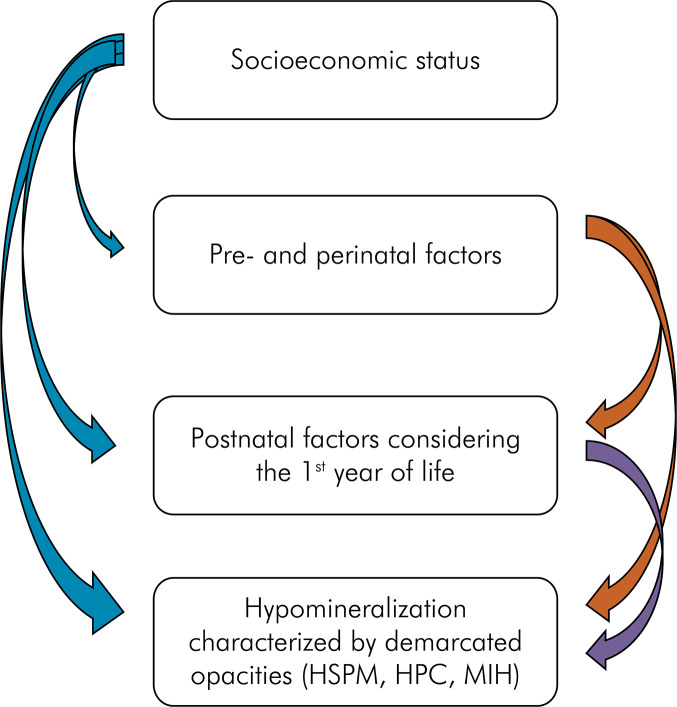
Hierarchical statistical analysis.

The collected data were entered into an Excel spreadsheet. Prevalence, distribution, and severity of the enamel defects were analyzed at the tooth level and the associated factors were evaluated at the individual level. The evaluation was performed according to hierarchical model analysis or multilevel modeling, whose aim is to evaluate whether the variables of interest have statistically significant interference in the other study variables. In Figure 2 shows the variables present in the hierarchical model. Socioeconomic data were utilized as the first variable, followed by prenatal, perinatal, and postnatal factors. Data on hypomineralization, which could be affected by the previously analyzed data, were considered the outcome variables.

Data tabulation (25% of the sample) was confirmed by a second researcher. Descriptive analysis, frequencies calculation, bivariate analysis, univariate and multivariate Poisson logistic regression with robust adjustment, and multivariate analysis with nonparametric resampling with Jacknife adjustment (95%CI) were performed using STATA 13.0 or higher (StataCorp. College StationUSA). Poisson logistic regression was chosen as the statistical test because it analyzes count data and its results show the factors that can predict the frequency of an event. Univariate, bivariate, and multivariate analyses were performed to simulate, as much as possible, a real-world scenario, given that, in real life, most outcomes have many predictors. The multivariate analysis with nonparametric resampling with Jacknife adjustment is commonly used to provide estimates of the bias and a standard error of a statistical test and it was performed to reduce the risk of bias due to the large number of zeros (sound tooth surfaces) in the data.

Four blocks of variables were considered for the statistical analysis. The factors analyzed for each block can be found in Figure 1: factors associated with socioeconomic variables, factors associated with prenatal and perinatal periods, factors associated with the postnatal period, and factors associated with HSPM/MIH.

Reference values for the second statistical analysis with p < 0.20 were included in the final model. In the multivariate analysis model, only one of the variables was included in the table. The other variable was the reference value with an IRP = 1 (incidence rate). The hierarchical analyses were performed according to the sequence illustrated in Figure 2, where all analyses of the presence of hypomineralization were performed using socioeconomic status and prenatal, perinatal, and postnatal factors as parameters.

The outcomes were assessed to establish the prevalence, distribution, and severity of hypomineralization characterized by demarcated opacities in primary teeth, assess which factors related to the child’s life and development could be related to the presence of hypomineralization, and correlate the presence of HSPM in primary dentition with the development of MIH in the permanent dentition.

## Results

A total of 2,102 participants were included in the study, and there were no dropouts. All sociodemographic data are presented in Table 1. The overall prevalence of hypomineralization in all primary teeth was 18.5%, with specific prevalence rates of 17% for HSPM and 6.7% for hypomineralized primary canines, as detailed in Table 2. In the entire sample, 81.5% of the children showed no developmental defects of enamel. Lower second molars were the most frequently affected, followed by upper second molars. Hypomineralization in canines was more prevalent in the upper arch. As for primary first molars, prevalence was 1.1% in the upper arch, with an even lower prevalence in the lower arch, while hypomineralization in incisors was nearly absent.

**Table 1:** Demographics and socioeconomic sample characteristics.

Participants (n = 2,102)	%
Children accompanied by their mothers	92.1
Sex	
Female	50.1
Male	49.1
Lived with parents (mother and father)	64
Mothers’ level of education	
Completed high school	60
Monthly family income	Less than three minimum wages

**Table 2:** Prevalence of hypomineralized primary teeth.

										
Tooth	55	54	53	52	51	61	62	63	64	65
%	8.7	1.1	3.3	0.1	0.2	0.1	0.1	3.1	1.1	9.3
Tooth	85	84	83	82	81	71	72	73	74	75
%	10.7	0.8	2.7	0.1	0	0	0.1	2.7	1.0	10.7

The number of affected teeth per child varied. Considering a prevalence of 18.5%, it was found that 4.5% of the cases involved only one second primary molar, 4.3% affected two teeth, and 3.1% affected three or four teeth. Interestingly, primary incisors and first molars were affected in only 0.4% of the cases, with no involvement of primary second molars and canines, and all second molars and canines exhibited demarcated opacities in only 0.3% of the cases. In terms of severity, the most prevalent defect was small white/cream opacities involving up to one-third of the tooth surface, which can be considered a mild defect. The occlusal surface of second molars was in some cases (1.8%) affected by larger and more severe defects such as posteruptive breakdown, atypical carious lesions, and restorations. The incisal surface of canines was mostly affected by posteruptive breakdown (0.3%). All information on distribution and severity of the lesions is presented in Table 3.

**Table 3:** Distribution and severity of hypomineralization on primaty teeth.

Tooth	55	53	63	65
Severity/Surface (%)	Mild/Buccal (3.6)	Mild/Buccal (2.4)	Mild/Buccal (2.1)	Mild/Buccal (3.5)
	Severe/Occlusal (1.4)			Severe/Occlusal (1.6)
Most prevalent defect/Surface (%)	White/cream opacities/Buccal (5.2)	White/cream opacities/Buccal (2.3)	White/cream opacities/Buccal (2.2)	White/cream opacities/Buccal (5.7)
	Posteruptive breakdown and atypical caries/ Occlusal (0.9)	Posteruptive breakdown/Incisal (0.3)	Posteruptive breakdown/Incisal (0.2)	Atypical caries/ Occlusal (1.0)
**Tooth**	**85**	**83**	**73**	**75**
Most prevalent defect/Surface (%)	White/cream opacities/Buccal (5.4)	White/cream opacities/Buccal (1.8)	White/cream opacities/Buccal (2.1)	White/cream opacities/Buccal (5.4)
	Posteruptive breakdown and atypical restorations/ Occlusal (1.4)	Posteruptive breakdown/Incisal (0.3)	Posteruptive breakdown/Incisal (0.1)	Atypical restorations/ Occlusal (1.9)
Severity/Surface (%)	White/cream opacities/Buccal (5.4)	White/cream opacities/Buccal (1.8)	White/cream opacities/Buccal (2.1)	White/cream opacities/Buccal (5.4)
	Posteruptive breakdown and atypical restorations/ Occlusal (1.4)	Posteruptive breakdown/Incisal (0.3)	Posteruptive breakdown/Incisal (0.1)	Atypical restorations/ Occlusal (1.9)
**Tooth**	**Primary first molars (54, 64, 74, 84)**		**Central (51, 61, 71, 81) and lateral incisors (52, 62, 72, 82)**	
Most prevalent defect/ Surface (%)	Only presented white/cream and yellow/brown opacities on the buccal and occlusal surfaces		Only presented white/cream opacities on the buccal surface	
Severity/ Surface (%)	Only presented mild defects on the buccal and occlusal surfaces		Only presented mild defects on the buccal surface	

**Figure 3:**
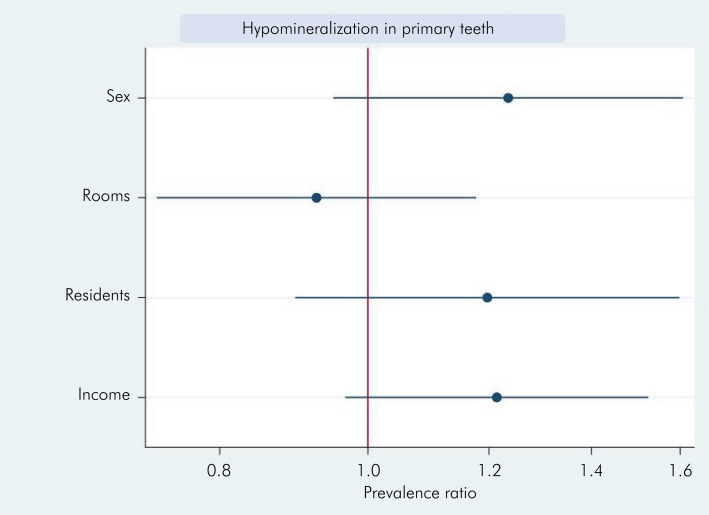
Prevalence ratio for hypomineralization in primary teeth considering socioeconomic factors.

When analyzing the factors associated with the development of hypomineralization in primary teeth, socioeconomic variables (block 1) were assessed first. A possible association was observed between the number of household members and the development of hypomineralization (p = 0.03). However the multivariate analysis showed a poor association between these factors (p = 0.223). The income variable (up to two minimum wages) was near-significant, with a p value of 0.95 in both analyses, but it is not possible to indicate any association (Figure 3 and Table 4). In all graphic representations, the red line represents the nullity line, indicating the null effect (relative risk equal to 1). Factors to the left of the line indicate protection, whereas those on the right side indicate risk.

**Table 4:** Poisson regression (bivariate and multivariate) associating the prevalence ratio for hypomineralization in primary teeth with variables related to socioeconomic factor.

Variables	Bivariate analysis			Multivariate analysis		
	IRP	p >|z|	95% CI	IRP	p >|z|	95%CI
Sex (male)	1.177	0.076	0.983–1.409	1.235	0.116	0.949–1.607
Number of rooms in the household (6–9)	1.159	0.099	0.973–1.380	0.926	0.529	0.728–1.177
Number of people living in the household (5–9)	1.394	0.003	1.120–1.736	1.197	0.223	0.896–1.598
Family income	1.182	0.095	0.971–1.438	1.214	0.095	0.966–1.525
Father’s level of education	0.986	0.895	0.796–1.220			
Mother’s level of education	0.946	0.640	0.750–1.193			

PD; pregnancy disease; SPD: smoking during pregnancy; CS: cigarette smoke.

**Figure 4:**
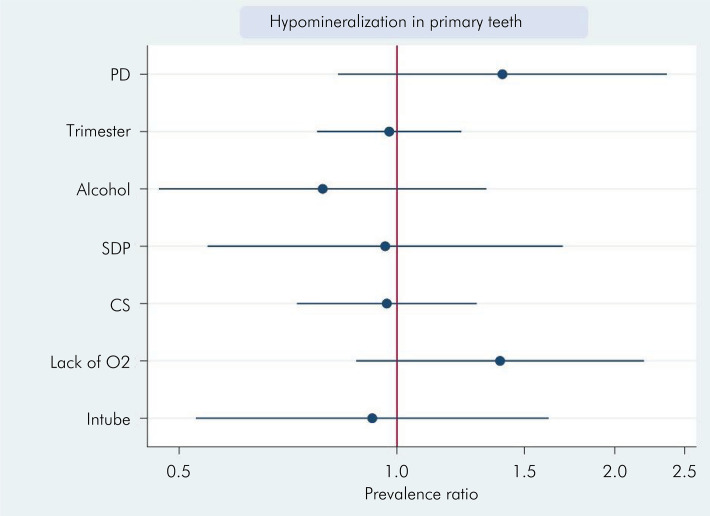
Prevalence ratio for hypomineralization in primary teeth considering prenatal and perinatal factors.

In the analysis of block 2 variables (prenatal and perinatal periods), the bivariate analysis identified three significant factors (p < 0.05): disease during pregnancy (p = 0.007), lack of oxygen at birth (p = 0.006), and intubation at birth (p = 0.041). In the multivariate analysis, none of the variables obtained statistically significant results (Figure 4 and Table 5).

**Table 5:** Poisson regression (bivariate and multivariate) associating the prevalence ratio for HDD with valiable related to prenatal and perinatal factors.

Variables	Bvariate analysis			Multivariate analysis		
	IRP	p >|z|	95%CI	IRP	p >|z|	95%CI
Prenatal	1.483	0.401	0.591–3.723	-	-	-
Disease during pregnancy	1.323	0.007	1.079–1.621	1.002	0.996	0.469–2.142
Pregnancy term in which the disease occurred	1.119	0.028	1.012–1.237	1.178	0.293	0.868–1.598
Folic acid	1.135	0.483	0.796–1.618	-	-	-
Drug use during pregnancy	0.824	0.716	0.290–2.339	-	-	-
Alcohol consumption during pregnancy	0.669	0.055	0.443–1.009	0.702	0.385	0.316–1.559
Smoking during pregnancy	0.695	0.097	0.452–1.068	0.506	0.211	0.174–1.473
Exposure to cigarette smoke during pregnancy	0.823	0.083	0.661–1.026	0.962	0.834	0.666–1.389
Mother’s age	1.018	0.870	0.820–1.264	-	-	-
Type of birth/delivery	0.901	0.277	0.746–1.088	-	-	-
Prolonged labor (more than 20 hours)	1.014	0.931	0.748–1.374	-	-	-
Gestational age at birth (in weeks)	0.924	0.640	0.663–1.287	-	-	-
Birth weight	0.891	0.446	0.663–1.198	-	-	-
Hypoxia at birth	1.543	0.006	1.133–2.101	1.547	0.139	0.867–2.758
Intubation at birth	1.454	0.041	1.016–2.081	0.659	0.314	0.292–1.486

**Figure 5:**
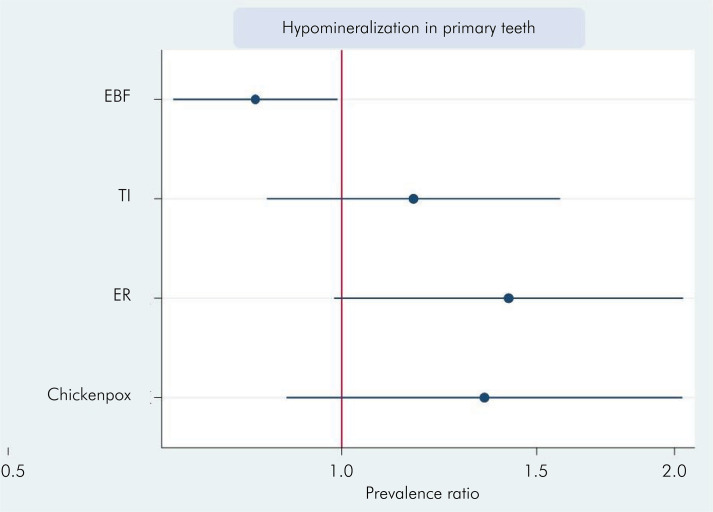
Prevalence ratio for hypomineralization in primary teeth considering factors related to the child’s first year of life.

EBF: exclusive breeastfeeding; TI: throat infection ER: ear infection.

Factors associated with the child’s first year of life (postnatal period - block 3) were examined in the bivariate analysis, revealing two significant variables: exclusive breastfeeding for the first 6 months of life (p = 0.040) and ear infection up to the first year of life (p = 0.032). The multivariate analysis demonstrated only exclusive breastfeeding for the first 6 months of life maintained its significance, indicating a lower prevalence of hypomineralization in primary teeth (Figure 5 and Table 6). Note that exclusive breastfeeding for the first 6 months of life was associated with a lower probability of hypomineralization in primary teeth, that is, exclusive breastfeeding for 6 months indicates that children are less likely to develop the enamel defect investigated in this study.

**Table 6:** Poisson regression (bivariate and multivariate) associating the prevalence ratio for HPT with variables related to the child’s first year of life.

Variable	Bivariate analysis			Multivariate analysis		
	IRP	p >|z|	95%CI	IRP	p >|z|	95%CI
Breastfed at birth	0.867	0.354	0.642–1.172	-	-	-
Exclusive breastfeeding (6 months)	0.836	0.040	0.705–0.992	0.836	0.040	0.705–0.992
Hospitalization up to 1 year of age	1.123	0.362	0.875–1.440	-	-	-
Fever up to 1 year of age	1.039	0.719	0.843–1.280	-	-	-
Antibiotic up to 1 year of age	1.136	0.229	0.923–1.398	-	-	-
Bronchitis	1.009	0.996	0.658–1.548	-	-	-
Bronchiolitis	0.923	0.656	0.649–1.312	-	-	-
Asthma	1.143	0.692	0.589–2.218	-	-	-
Pneumonia	1.325	0.162	0.894–1.964	-	-	-
Rhinitis	0.889	0.605	0.570–1.388	-	-	-
Throat infection	1.231	0.133	0.939–1.615	1.161	0.097	0.857–1.574
Ear infection	1.433	0.032	1.030–1.992	1.415	1.88	0.984–2.035
Sinusitis	1.026	0.926	0.598–1.760	-	-	-
Anemia	1.308	0.453	0.649–2.635	-	-	-
Heart condition	0.518	0.328	0.138–1.936	-	-	-
Cholesterol	0.778	0.639	0.273–2.220	-	-	-
Diabetes	1.041	0.949	0.300–3.613	-	-	-
Chickenpox	1.445	0.076	0.962–2.173	1.346	0.156	0.892–2.032
Measles	0.577	0.561	0.091–3.676	-	-	-
Atopic dermatitis	1.277	0.290	0.812–2.008	-	-	-
Allergy	0.724	0.362	0.361–1.450	-	-	-
Congenital disease	0.823	0.714	0.290–2.336	-	-	-

**Figure 6:**
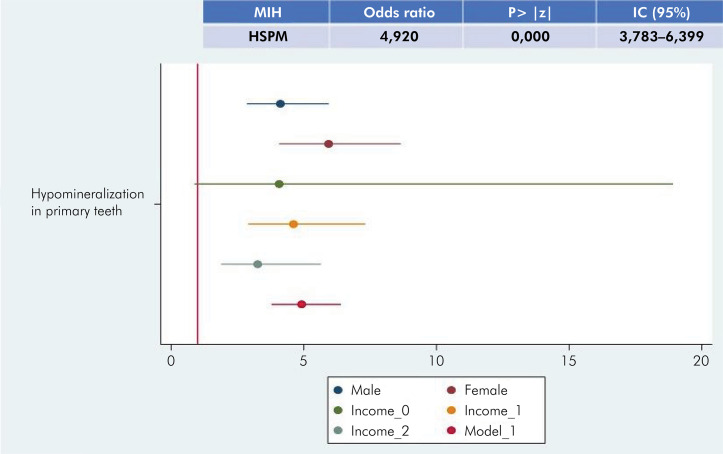
Logistic regression of the association between HSPM and MIH and odds ratio between the association of HSPM and MIH considering socioeconomic factors.

The association between HSPM and MIH was analyzed using univariate and multivariate Poisson logistic regressions and bivariate analysis. It was found that children with HSPM are approximately five times more likely to have MIH (OR =4 .9). Logistic regression was also performed, considering socioeconomic factors (sex and family income). Regardless of family income and sex, the association between HSPM as a predictor of MIH remains significant (Figure 6). No difference was observed between sexes, as neither offered protection against MIH, and males have a slightly lower risk when compared to females (Figure 6).

Considering family income, all factors indicated risk. Family income 0 (unemployed and family allowance) was the only result that was not statistically significant, which slightly crossed the red line and was at odds with the other results obtained for other income values. Family income values 1 and 2 (up to two minimum wages and more than two minimum wages) indicated risk for the association between HSPM and MIH, with family income 1 having a higher risk when compared to family income 2. To ensure data accuracy, 20% of the tabulation and results were re-evaluated and checked by different professionals.

## Discussion

The prevalence of hypomineralization in all primary teeth (18.5%) was higher than 12.5%^18, 19^ and similar to 16.8%^20^ across all Brazilian studies. In Spain, the prevalence was 1.7%,^21^and in Tanzania, 5.0%.^22^ Some results differ from those found in the present study, and this can be explained by the different characteristics of the sample and by the use of a different diagnostic index, as all studies used the modified DDE index, which is not a specific index to classify hypomineralization characterized by demarcated opacities, leading to diagnostic bias.

The prevalence of HSPM reported worldwide varies widely. Similar prevalence rates have been reported, such as 14.6%^15^ and 18.9%^23^in Spain and 14.1% in Australia.^24^ However, studies conducted in other countries have shown lower HSPM prevalence rates , as low as 6.6% in Iraq,^2^ 5.2% in Canada^,13^ and 4.9% in the Netherlands.^4^ The wide variation in prevalence observed worldwide can be explained by the different populations evaluated, their characteristics, and the environmental factors to which they are exposed. In general, national and international studies have pointed out that the prevalence of HSPM does not exceed 20%.

The distribution of hypomineralization in this study corroborates previous findings, indicating that tooth 85 exhibits the highest prevalence among all primary teeth,^18^ but our findings differ from those of other studies,^4,8,15^ which identified tooth 55 as the most frequently affected. While the literature lacks an explanation for why second molars are commonly the most affected by HSPM, it does address why second molars are more affected by carious lesions.^25^ Second molars are very likely to be the most affected by enamel defects among primary teeth because most of their crown mineralization occurs in the first year of life,^26^ when children are more susceptible to factors that can interfere with enamel formation.

The severity of hypomineralization characterized by demarcated opacities is influenced by the progression of the defect. White/cream demarcated opacities are considered mild, evolving into posteruptive breakdown, carious lesions, or restoration in severe cases.^5,23^ The most prevalent type of hypomineralization in the present study was white/cream demarcated opacities, followed by yellow/brown demarcated opacities and posteruptive breakdown, thus corroborating the findings of previous studies.^4,8,7,13,24,26,27^

The etiology of hypomineralization characterized by demarcated opacities in primary teeth is still uncertain. The literature indicates that systemic insults occur during dental enamel formation, influencing the development of these defects, mainly due to the increase in body temperature, which directly interferes with amelogenesis.^3,16,17^ The literature highlights some studies on etiological factors, including systematic reviews,^4,5^ longitudinal studies,^6,18^and cross-sectional studies.^2,7,8^

The factors reported in the literature served as a parameter for the development of the questionnaire used in the present study. However, no association was observed between any of these factors and hypomineralization in primary teeth. This finding is also important as it shows that many factors previously associated in the literature with hypomineralization in primary teeth were not observed in the analyzed sample. This may be explained by the characteristics of the sample or by time bias. As this is a cross-sectional study, data were collected at a single point in time, with no follow-up of children since pregnancy.

On the other hand, the present study found that exclusive breastfeeding for 6 months is associated with a lower probability of developing hypomineralization, with a statistically significant result, which corroborates and complements some other studies that suggested a possible association but did not have statistically significant results.^29-31^ A study carried out in Brazil^32^ indicated an association between children breastfed for less than 6 months and the development of enamel defects, but included all defects in its analysis (demarcated opacity, diffuse opacity, hypoplasia, and amelogenesis imperfecta), i.e., not only those characterized by demarcated opacities, as done in the present study.

Exclusive breastfeeding for 6 months has biological plausibility and is considered a protective factor for numerous diseases, reducing the incidence of respiratory and gastrointestinal infections,^33^ in addition to having numerous other benefits for child health and development, such as fewer allergies, lower disease occurrence, and better nutrition.^34,35^ The decision not to breastfeed a child has important long-term effects on their health, nutrition, and development, and on maternal health as well.^36^ As exclusive breastfeeding reduces the chances of diseases that can cause fever and the need for medication – factors that have already been associated with the development of hypomineralization – this may explain why the exclusive breastfeeding for 6 months is associated with lower probability of hypomineralization, given that healthier children are less prone to developmental defects of enamel.^36-40^

According to the literature, the presence of hypomineralized second primary molars may be a predisposing factor for the development of MIH in permanent teeth.^10,12^ The present study corroborates this finding, showing that children with HSPM are 4.9 times more likely to have MIH, which is in line with other studies,^10,11^ which reported odds ratios of 4.4 and 6 for the development of MIH. Note that the absence of this defect in the deciduous dentition does not prevent the development of MIH.

Another important aspect to be considered is that the predictive factor associated with HSPM underscores the need for monitoring these patients throughout primary and permanent dentition and conducting regularreassessments.^12^ It is extremely important to monitor the eruption of permanent teeth so that it is possible to prevent pain, sensitivity, posteruptive breakdowns, carious lesions, and restorations associated with demarcated opacities, thus preventing the progression of the lesion and its impact on children’s oral health.

The present study also analyzed the association between sex, family income, and the presence of HSPM and MIH, a type of analysis that had not been performed in previous studies. The findings, regardless of these factors, indicate that the association between the presence of HSPM and MIH remains statistically significant. No difference was observed between sexes, indicating that neither sex offers protection against the association between HSPM and MIH, with males presenting a slightly lower risk when compared to females.

When analyzing family income and the association between HSPM and MIH, lower income levels are correlated with a higher probability ratio for the development of MIH in children with HSPM. In this analysis, one of the income categories (unemployment and family allowance) did not indicate statistical significance, which may be due to the low incidence of this variable, considering that most of the sample had a family income of up to two minimum wages. Low income is usually associated with lower quality of life, poorer education, and difficulty in accessing quality health services and information, all of which may account for the increased incidence of HSPM/MIH.^1,6,7^

Limitations of this study include time bias, due to the cross-sectional design, with data collection at a single point in time. This may introduce recall bias regarding pregnancy and the child’s first year of life. In addition, the study can identify associations, not causal relationships. Another limitation is the sample analyzed which, although representative of the local population of Diadema/SP, did not include children from all socioeconomic backgrounds. The data were collected from local PHCs, where most of the children come from a socioeconomic background in which their families earn up to two monthly minimum wages. The final limitation concerns the larger number of healthy teeth (without defects) in the sample, resulting in an inflated number of zeros (representing healthy teeth) in the database which could have caused bias. This was addressed, however, by performing nonparametric resampling with Jacknife adjustment for statistical analysis.

Furthermore, the study provides valuable insight into the prevalence and risk factors associated with hypomineralization in primary teeth, particularly in Brazilian children, and underscores the importance of exclusive breastfeeding as a potential protective factor, corroborating the findings of previous studies.^29-32^ Despite the limitations related to the cross-sectional design and sample characteristics, the statistical rigor applied, such as nonparametric resampling, ensures the robustness of the results. These findings contribute to the existing literature by supporting the connection between hypomineralization in primary and permanent teeth, reinforcing the need for early monitoring and preventive care for affected children. Future studies with diverse and longitudinal samples are needed to further elucidate causative factors and strengthen preventive strategies for enamel hypomineralization.

## Conclusion

This study found that the prevalence of HSPM is consistent with global trends, with overall hypomineralization in primary teeth presenting moderate rates. While second primary molars and primary canines were the most affected tooth elements, other primary teeth also showed signs of hypomineralization, albeit at lower frequencies. Most lesions were mild and of limited extent.

Exclusive breastfeeding for the first six months of life was associated with a lower likelihood of developing hypomineralization characterized by demarcated opacity in primary teeth, suggesting a protective effect. Furthermore, children with HSPM were found to be five times more likely to develop MIH in permanent teeth, regardless of sex. Additionally, lower family income was correlated with a higher risk of MIH among children with HSPM, highlighting socioeconomic influences on oral health outcomes.
